# Parasite Pressures on Feral Honey Bees (*Apis mellifera sp.*)

**DOI:** 10.1371/journal.pone.0105164

**Published:** 2014-08-15

**Authors:** Catherine E. Thompson, Jacobus C. Biesmeijer, Theodore R. Allnutt, Stéphane Pietravalle, Giles E. Budge

**Affiliations:** 1 University of Leeds, Woodhouse Lane, Leeds, United Kingdom; 2 The Food and Environment Research Agency, Sand Hutton, York, United Kingdom; Ghent University, Belgium

## Abstract

Feral honey bee populations have been reported to be in decline due to the spread of *Varroa destructor*, an ectoparasitic mite that when left uncontrolled leads to virus build-up and colony death. While pests and diseases are known causes of large-scale managed honey bee colony losses, no studies to date have considered the wider pathogen burden in feral colonies, primarily due to the difficulty in locating and sampling colonies, which often nest in inaccessible locations such as church spires and tree tops. In addition, little is known about the provenance of feral colonies and whether they represent a reservoir of *Varroa* tolerant material that could be used in apiculture. Samples of forager bees were collected from paired feral and managed honey bee colonies and screened for the presence of ten honey bee pathogens and pests using qPCR. Prevalence and quantity was similar between the two groups for the majority of pathogens, however feral honey bees contained a significantly higher level of deformed wing virus than managed honey bee colonies. An assessment of the honey bee race was completed for each colony using three measures of wing venation. There were no apparent differences in wing morphometry between feral and managed colonies, suggesting feral colonies could simply be escapees from the managed population. Interestingly, managed honey bee colonies not treated for *Varroa* showed similar, potentially lethal levels of deformed wing virus to that of feral colonies. The potential for such findings to explain the large fall in the feral population and the wider context of the importance of feral colonies as potential pathogen reservoirs is discussed.

## Introduction

The feral honey bee colonies of the UK were thought to have been severely reduced by the arrival of the *Varroa* mite in 1992 [1], [2], which also caused severe losses in the managed population [3]. However, anecdotal reports have spoken of a recent resurgence in the feral honey bee population, and have suggested this could be due to the evolution of an adapted host parasite relationship [3]–[5].

*Varroa destructor* is an ectoparasitic mite that has both direct and indirect impacts on honey bee health. The mite causes direct damage to the developing honey bee larvae and pupae by sucking their haemolymph and reducing their hatching weight [5]. Bees parasitized in this way usually begin foraging earlier and have a significantly reduced lifespan which may be due to decreased learning abilities, impaired ability to navigate and consequently a lower probability of returning to the colony [5]. Indirect effects of *V. destructor* are termed varroosis, whereby the *Varroa* mite acts as a vector for a variety of honey bee viruses, most notably deformed wing virus (DWV) [6].

Before the occurrence of *Varroa* mites, bee viruses were generally considered a minor problem to honey bee health [5]. Recently, however, Genersch (2010) found DWV and ABPV to be significantly related to German winter colony loss, while Highfield et al (2009) attributed 67% of overwintering colony loss in Devonshire to DWV [7], [8]. Indeed varroosis is now considered to be the most destructive disease of honey bees worldwide [5], [6] and a major cause of winter colony loss [9], [10].

Beekeepers control *Varroa* levels in colonies using synthetic acarides, organic acids, essential oils and a wide variety of management techniques, which has helped to improve survival rates [5], [11]. It was assumed, given the early large scale losses of untreated managed populations that feral, and thus untreated colonies, must quickly succumb to varroosis, although no evidence was ever presented to support this theory. However, it has been also suggested that sufficient time has passed since the first exposure to *Varroa* mite infestation to allow selection pressure to act on bee populations, and that feral honey bee populations are starting to rebound [4], [12]. Indeed, shorter-term selective breeding of managed colonies for ‘*Varroa* resistance’ has been shown to lower *Varroa* numbers in some colonies [4], [13], [14]. In the Arnot Forest, in the US, feral colonies were found to be at the same density in 2007 as in pre *Varroa* times in 1978 [13], [15]. Untreated colonies in France and Sweden were also used for a ‘live and let die experiment’, where colonies that survive without *Varroa* treatment are subsequently selected for honey production in an attempt to create a race that is both *Varroa* tolerant and economically attractive [13], [14], [16]. There is also a strong body of evidence from laboratory and field studies to show rapid evolutionary responses to selection pressures in other insect–pest interactions, such as in the case of *Drosophila melanogaster* populations and the parasitoid *Asobara tabida* [17], [18].

If found to be coping with varroosis in the absence of active management, feral honey bee colonies could act as important genetic stocks from which to improve breeding efforts for mite tolerant managed honey bees [5], [19]. Alternatively, feral nests could simply represent escaped swarms from managed colonies that could present a risk to the managed population by harbouring disease agents and re-infecting managed stocks [20], [21].

Controlling communicable disease in managed honey bee populations is a challenge, given honey bees can move disease agents over great distances. Adult bees can be used to infer the infection state of a colony, allowing the disease state of a colony to be determined without the need for a destructive sample of brood [22]. This is the first time these methods have been used to measure the pathogen burden of the UK's feral honey bee population, and the first study to compare feral pathogen levels with those of local managed colonies.

## Methodology

### Site selection

Feral honey bee colonies were located by engaging the beekeeping community and the general public using several methods; (1) emails to beekeeping associations (both the main BBKA secretary, but also secretaries of regional beekeeping associations); (2) notes on applicable internet forums such the natural beekeeping forum; and (3) an article in BeeCraft, a popular monthly beekeeping magazine [23]. Respondents were able to report their colony by email, letter, or using a bespoke website (www.honeybeeproject.co.uk).

Locations of feral colonies were selected based on a good history of activity at the nest site (1 year minimum) thus avoiding the inclusion of new swarms with no history of survival. Sites that were inaccessible were not selected for safety reasons. The managed apiary nearest the feral site was identified using a national beekeeping register called BeeBase (see www.nationalbeeunit.com) and samples of adult honey bees were collected from feral and managed colonies on the same day in Spring 2010. At the same time each beekeeper was asked whether they actively controlled *Varroa*. Unfortunately it was impossible to carry out *Varroa* screening at the colony site as most cavities were inaccessible and *Varroa* mites were only seldom seen on adult workers collected at the colony entrance.

All sites surveyed were on private property. Permission was sought from the individual land owners for house and garden sites, and the National Trust for parks and estates. Field studies did not involve protected or endangered species.

### Nucleic acid extraction

Approximately 60 foraging *A. mellifera* adults were collected from each colony and stored for use in 100% ethanol at −70°C [24]. Twenty-four bees from each of the 34 paired colonies were selected. Whole bees were washed in molecular grade water, and individually disrupted with 2.3 mm silica beads in bead beater at 8000 g for 30 seconds (Precellys). Total DNA and RNA was extracted from each worker bee using a 10% Chelex solution with TE buffer. After disruption, 800 µl of 10% Chelex solution was added to each crushed bee residue. The solution was heated to 95°C for 5 minutes then centrifuged at 8000 g for a further 5 minutes. The upper aqueous layer was removed (200 µl) before centrifugation at 8000 g for 5 minutes, with the removal of 150 µl of the upper aqueous DNA. Finally, 20 µl of extract from each individual bee was pooled per colony [8]. Colony extractions were divided and purified to optimise RNA [25] and DNA recovery [26]. Briefly, 300 µl of extract was mixed with 300 µl of 24∶1 chloroform: IAA solution and spun at 8000 g for 10 minutes. For RNA, 100 µl of the upper aqueous layer was transferred into a fresh tube containing 100 µl of 4 M LiCl and samples were mixed well and left overnight. For DNA 100 µl of the upper aqueous layer was transferred into a fresh tube containing 50 µl of 5 M NaCl and 100 µl isopropanol. Next both DNA and RNA samples were vortexed and centrifuged for 10 minutes 8000 g. The remaining salt and ethanol was decanted and the pellet was washed with 500 µl of 70% ethanol by spinning for 4 minutes. The ethanol was decanted and the pellet dried in a heated vacuum for 5 minutes at medium heat. Dried pellets were re-suspended in 150 µl of 1× TE buffer and frozen at −20°C until required. Extraction blanks were created during this process using the same reagents.

### Parasite testing

Colony extracts were tested for the presence of *Acarapis* mites, black queen cell virus (BQCV), chronic bee paralysis virus (CBPV), deformed wing virus (DWV), *Nosema apis*, *Nosema ceranae*, *Melissococcus plutonius*, *Paenibacillus larvae*, *Slow paralysis virus* (SPV) using established protocols (Table 1). Real-time reactions were set-up using TaqMan chemistry using PCR core-reagent kits (Applied Biosystems, Branchburg, New Jersey, USA), according to the manufacturer's protocols. Reactions (25 µl) were comprised of 2.5 µl of buffer (Buffer A), 5.5 µl MgCl_2_ (25 nM), 2 µl dNTP, 1 µl of forward and reverse primers, 0.5 µl of probe, 0.125 µl Taq polymerase, 0.125 µl MMLV (RT reactions only) and 5 µl of DNA extract (parasites) or RNA (viruses). All Taqman probes were specific and covalently labelled with a reported dye (FAM) at the 5′ end and with a quencher dye (TAMRA) at the 3′ end (Table 1). Samples were run in triplicate reactions with template positive and extraction blanks.

**Table 1:** Primers used in this study.

Target	Primer name	Sequence (5′-3′)
Acarapis spp.^5^	*Acarapis F1*	GCCATAAGACATCACTATTCT
	*Acarapis R1*	TCATTTAAACTTCATGATACTCTCAATCAG
	*Acarapis T*	TGCGCAATGCAACTAGTCCTCTAAAGACTAGTTTC
Black queen cell virus 1	*BQCV 8195F*	GGTGCGGGAGATGATATGGA
	*BQCV 8265R*	GCCGTCTGAGATGCATGAATAC
	*BQCV 8217T*	TTTCCATCTTTATCGGTACGCCGCC
Chronic bee paralysis virus^1^	*CBPV 304F*	TCTGGCTCTGTCTTCGCAAA
	*CBPV 371R*	GATACCGTCGTCACCCTCATG
	*CBPV 325T*	TGCCCACCAATAGTTGGCAGTCTGC
Deformed wing virus^1^	*DWV 958F*	CCTGGACAAGGTCTCGGTAGAA
	*DWV 9711R*	ATTCAGGACCCCACCCAAAT
	*DWV 9627T*	CATGCTCGAGGATTGGGTCGTCGT
Elongation factor 1^2^ (Internal control)	*EF1 F*	CTGGTACCTCTCAGGCTGATTGT
	*EF1 R*	GCATGCTCACGAGTTTGTCCATTCT
	*EF1 T (TAMRA)*	TGCTTCGAACTCTCTCCAGTACCAGCAGCAACA
*Nosema apis* 5	*N apis F1*	ATTTACACACCAGGTTGATTCTGC
	*N apis R1*	TGAGCAGTCCATCTTTCAGTACATAGT
	*N apis MGB*	TGACGTAGACGCTATTC
*Nosema ceranae* 5	*Nosema c1 83G F*	TTG AGA GAA CGG TTT TTT GTT TGA G
	*Nosema c1 974 R*	TTC CTA CAC TGA TTG TGT CTG TCT TTA A
	*Nosema c1 865 T*	ATA ATA GTG GTG CAT GGC CGT TTT CAA TGG
*Melissococcus plutonius* 3	*EFB F*	TGT TGT TAG AGA AGA ATA GGG GAA
	*EFB Rev2*	CGT GGC TTT CTG GTT AGA
	*EFBProbe*	AGA GTA ACT GTT TTC CTC GTG ACG GT
*Paenibacillus larvae* 5	*Pl_R24_468F*	TCCCCGAGCCTTACCTTTGT
	*Pl_R24_538R*	ACCTACGAACTTGACGCTGTCCT
	*Pl_R24_489T*	TGCTCATACCCGGTCAGGGATTCGA
*Slow paralysis virus* 4	*SPV 8383F*	TGATTGGACTCGGCTTGCTA
	*SPV 8456R*	CAAAATTTGCATAATCCCCAGTT
	*SPV 8407T (TAMRA)*	CCTGCATGAGGTGGGAGACAACATTG
*Sacbrood virus* 1	*SBV 311F*	AAG TTG GAG GCG CGy AAT TG
	*SBV 380R*	CAA ATG TCT TCT TAC dAG AGG yAA GGA TTG
	*SBV 331T*	CGG AGT GGA AAG AT

The 5′-terminal reporter dye for each *TaqMan* probe was 6-carboxyfluorescin (FAM) and the 3′ quencher was tetra-methylcarboxyrhodamine (TAMRA) or Minor groove binding (MGB) as indicated.

1 -[49].

2 -[1].

3 -[48].

4 -[50].

5 -[51].

Reactions were run on an ABI Prism 7900HT (Applied Biosystems) with real-time data collection. Reverse transcription was performed at 48°C for 30 minutes, followed by denaturing and enzyme activation at 95°C for 10 minutes. This was followed by 40 cycles of denaturing at 95°C for 15 seconds and a combined annealing and extension step for 60 seconds at 60°C. Fluorescence values, amplification plots and threshold cycle (C_t_) values were calculated using SDS 2.2 (Applied Biosystems).

### Quantification of PCR results

The relative amount of DWV, BQCV, *N. apis* and *N. ceranae* were analysed using the comparative C_t_ method [27] using the parasite specific assay as the target and Elongation Factor 1 (EF1) as the reference assay (Table 1). The use of this gene as an internal control is established [28], [29]. A survey of control genes, suggested that variation among the samples using four different references genes for normalization, including EF1, was rather small and did not drastically change the target gene expression profiles between samples [30]. PCR efficiencies between target and reference assays was compared by diluting a known positive sample 1∶10 through 6 levels. Efficiencies above 90% with an R2 above 0.98 indicated data [31].

### Wing morphology

Wing morphometry data was gathered for all colonies where more than 50 useable right forewings were available. Wings were removed from the bee and placed under a glass on an Epson Perfection V300 Photo scanner. Images were scanned at 4800dpi resolution using positive film strip mode. DrawWing software version 0.45 was used as the best example of modern wing morphometry, to record the cubital, hantel and discoidal shift index [32], [33]. Landmarks were placed by eye were DrawWing failed to correctly identify venation junctions on wings with slight damage to the tips. DrawWing outputs were transferred to Morphplot version 2.2 [34] and the average cubital, hantel and discoidal shift index calculated for each colony.

### Statistical analysis

Data for the four most commonly found parasites were analysed by Restricted Maximum Likelihood (REML) to account for the paired structure of the data using GenStat 14.1 [35]. The pairs were included in the model as a random effect whilst the treatment of interest (managed vs feral colonies) was included as a fixed effect. Further, the data were log-transformed to correct for right skew.

Log DWV levels were compared between three groups: feral (untreated), managed (treated) and managed (untreated) by analysis of variance. Individual treatments were compared using post-hoc test and a Tukey adjustment for multiple comparisons. This analysis was carried out in R version 3.0.0 [36].

Cubital index, discoidal shift angle and hantel index was analysed for feral and managed colonies. These index values were log-transformed to account for a right skew, and analysed by multivariate analysis of variance, using R version 3.0.0.

## Results

### Paired samples

In total, 100 reports of feral colonies were received and 60 were visited in Spring 2010. Of those visited, 34 feral colonies were sufficiently accessible to collect foraging bees and each was successfully paired with a nearby site containing a managed honey bee colony. Pairs were an average of 1.4 km apart, with a maximum of 10.9 km.

### Parasite testing

C_t_ values for positive controls and buffer blanks were checked initially, to ensure correct functioning of the master mix and an absence of contamination. All colony samples tested negative for SPV and *P. larvae*, *M. plutonius*, SBV, *Acarapis* spp. and CBPV were detected at low prevalence (Table 2).

**Table 2:** Results from testing nucleic acid preparations for parasites with fewer than five positive observations.

Disease	Feral Positives	Managed Positives
**Acarapis spp.**	1	4
**CBPV**	3	4
**M. plutonius**	0	1
**P. larvae**	0	0
**SBV**	1	0
**SPV**	0	0

**Figure 1:**
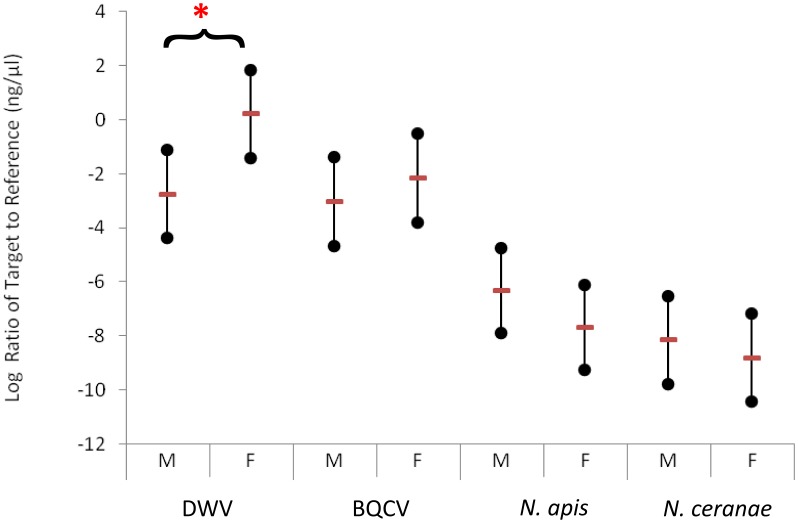
The Restricted Maximum Likelihood model estimates for the four most commonly found pathogens. Predictions are on the log scale with 95% confidence intervals. * denotes a significant different between paired managed (m) and feral (f) colonies. Analyses are done separately for each pathogen and not between pathogens.

**Figure 2:**
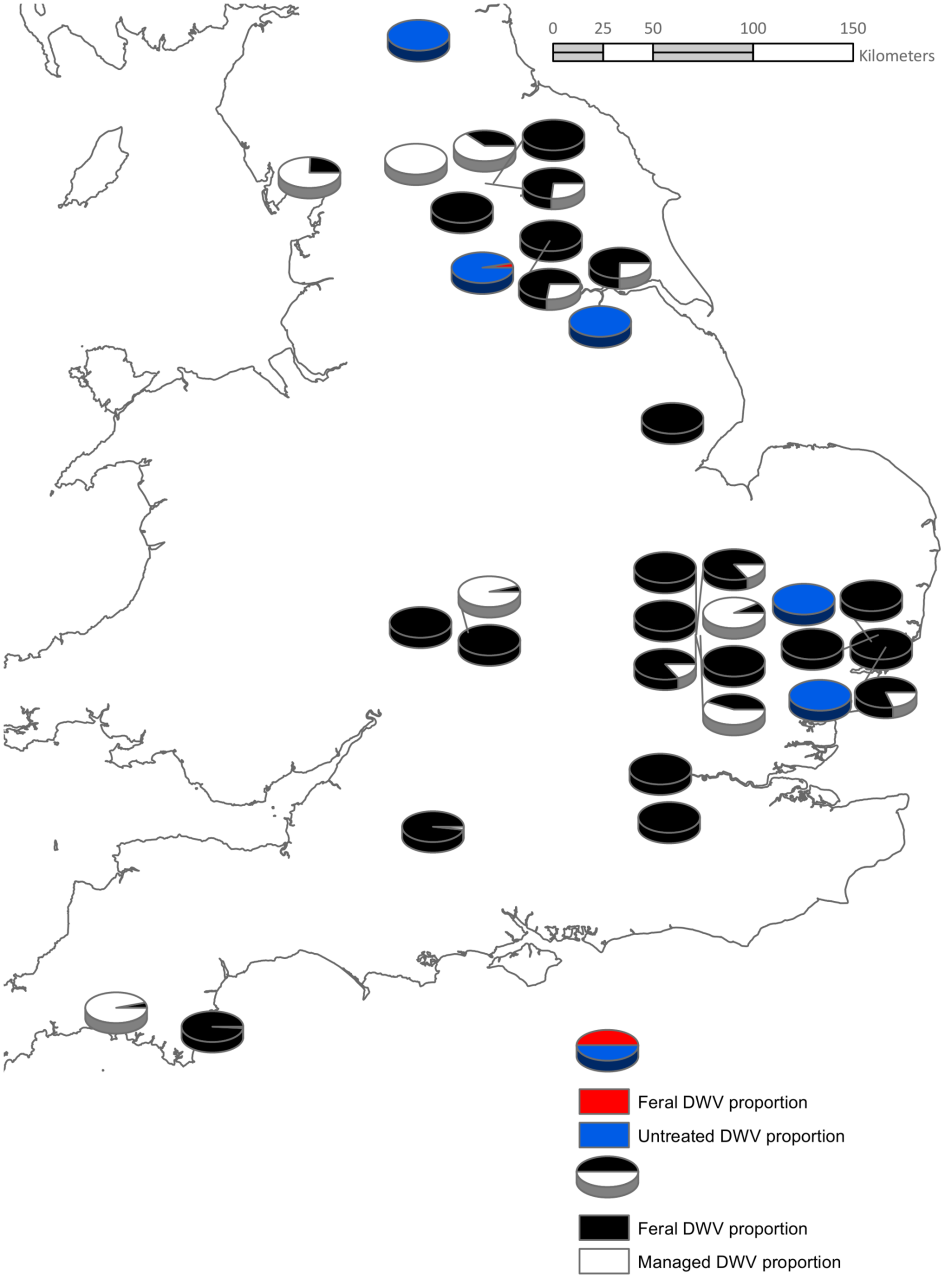
The proportion of DWV between pairs of either feral/managed or feral/untreated managed colony pairs. The pie charts are split into two groups: the red fill indicates feral colony DWV levels alongside the blue fill for managed colonies where no Varroa treatment was used. The black fill indicates feral colony DWV levels alongside the white fill of Varroa treated managed colonies.

**Figure 3:**
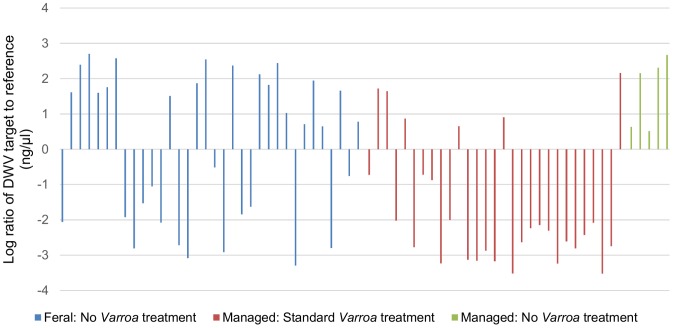
The effect of *Varroa* treatment on managed and feral colony log DWV levels separated by treatment. Blue indicates feral colonies untreated for *Varroa*. Red indicates managed colonies undergoing standard *Varroa* treatment (i.e. dosing with Varroacide one to two times per year). Green indicates managed colonies where no *Varroa* treatment was used.

All colonies were positive for DWV, BQCV, *N. apis* and *N. ceranae*. Similar PCR efficiencies (between 92% and 107%) were achieved for target assays and 96% for the reference assay allowing quantitative interpretation of the commonly detected pathogens [37]. There was no significant difference in the amount of *N. apis* (F = 1.70, d.f = 1, 33, p = 0.20,), *N. ceranae* (F = 0.52, d.f. = 1,33, p = 0.48) or BQCV (F = 1.11, d.f. = 1,33, p = 0.30) between feral and managed colonies. However, the amount of DWV was significantly different between managed and feral colonies (F = 6.41, d.f = 1,33, p = 0.016) (Fig. 1, 2 and 3).

**Figure 4:**
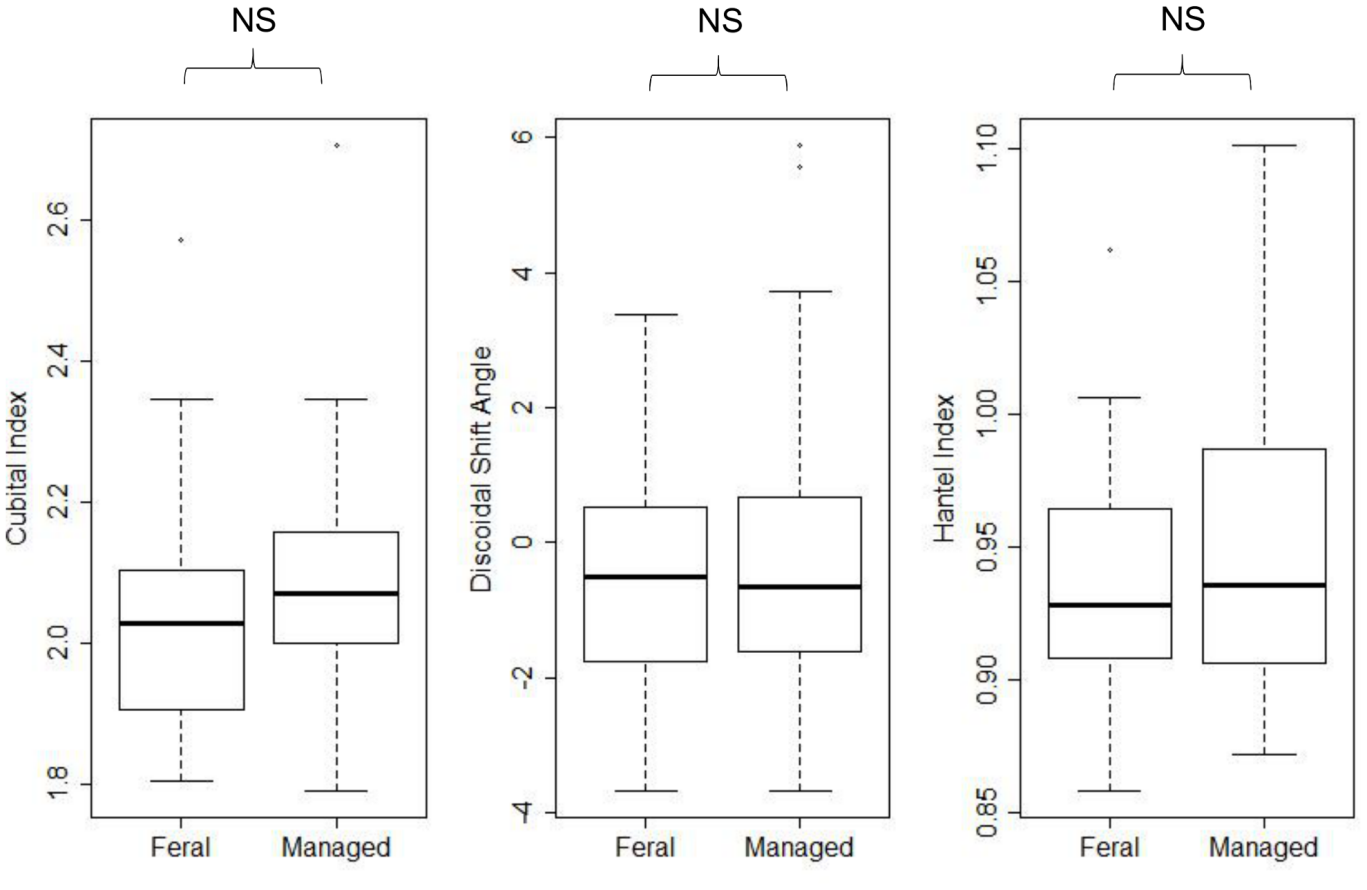
The morphometric results for cubital index, discoidal shift angle and hantel index for feral and managed colonies. There was no significant difference between feral and managed colonies for any of the three morphometric indices.

In total, usable right wings were obtained in sufficient number from 56 colonies (28 feral and 28 managed) to obtain wing morphometry metrics. There was no significant difference between wing morphometric indices for managed (n = 28) and feral colonies (n = 28) (approx. F = 0.43; df = 6, 104; p = 0.86, Fig. 4).

## Discussion

We present novel data to describe the parasite burden of feral honey bee colonies and report that three of the major parasites show similar intensity in both managed and feral adult honey bee populations. Crucially, the levels of DWV were far higher (Fig. 1) in feral colonies compared to managed colonies, which could reflect the absence of *Varroa* control in feral colonies.

Interestingly managed colonies not treated for *Varroa* contained similar virus levels to feral colonies (Fig. 3). DWV is the most prevalent known virus in managed honey bee colonies in Europe and an increasing proportion of viruliferous *Varroa* mites has been linked to reduced colony survival [10]. Colonies with high levels of DWV show evidence of a scattered brood nest, crippled bees, loss of coordinated social behaviour such as hygienic behaviour, queen attendance and a rapid decline in the colony's bee population [5], [6], [10], [38]. The extremely high values of DWV found in feral and untreated managed colonies would be expected to lead to colony mortality [8], however it is not clear whether feral colonies have novel mechanisms to resist such high levels of DWV. Whilst it is not possible to rule out increased *Varroa* tolerance or a balanced host parasite relationship in feral nests [3]–[5], the development of alternative coping strategies to mitigate the high DWV levels detected seems unlikely.

For the first time, we also present results that suggest the venation of the forewings of feral honey bees is not distinguishable from managed honey bees using three different morphometric measures (Fig. 4). Both the native honey bee of the British Isles (*Apis mellifera mellifera*), and the dominant European races (*A. m. carnica*, *A. m. iberica* and *A. m. ligustica*;[39]) all have well characterised morphometry. Whilst wing morphometry is often used to determine honey bee race, and has been criticised for being over reliant on the wing section of the genome [40], it gives us a useful first assessment of the apparent genetic similarity between feral and managed colonies [41]. Such morphological similarities suggest that the feral population may simply be a consequence of escapees from the managed population. Anecdotal increases in feral populations could simply reflect a combination of a recent increase in the popularity of beekeeping leading to a higher number of novice beekeepers who are likely to allow their colonies to swarm, and an increasing proportion of let-alone beekeepers who actively encourage natural honey bee behaviours like swarming [42]. A workable level of *Varroa* tolerance is keenly sought by UK beekeepers, but abandoning *Varroa* treatment without ensuring colonies have evolved a natural resistance to the mite, or without virus-free *Varroa*, could leave colonies dangerously exposed, particularly in areas of high beekeeping density [7], [43]. Indeed the geographic proximity, and lack of large, remote and untreated honey bee populations may prohibit meaningful breeding programs for *Varroa* resistance in England and Wales. Instead, a more gradual approach of selective breeding for *Varroa* tolerance is likely to lead to the improvements in resilience that global apiculture requires [44].

More BQCV was found in feral colonies compared to managed colonies, but this difference did not reach statistical significance (Fig. 1). *V. destructor* has been reported to vector this virus [37], so it is not inconceivable that this slight increase is also linked to mite parasitism. Nosemosis C is an emerging disease in Europe caused by *N. ceranae* [45], yet is found to be well established in feral colonies. This result could suggest a degree of parasite perturbation between feral and managed populations [46]. One can hypothesise that feral honey bees are exposed to fewer stressors in the form of beekeeper manipulation e.g. direct damage to comb and propolis, death of bees during beekeeper activity, cross contamination between hives, honey removal, pollen harvesting etc. [16]. In this study only one colony was positive for the causative agent of European foulbrood, a disease sometimes linked to stress [47], and this was a managed colony. The prevalence of EFB is low in the UK [48], so the small sample size makes it impossible to draw any meaningful conclusions about the true level of foulbrood infection in the UK's feral colonies. However, we can conclude that the sampled feral population contained many of the parasites commonly found in managed populations. Therefore, our study provides no evidence that feral-nests reduce parasite load compared to managed nests. However, our results do not address the consequences of the measured pathogen burden in feral nests, and it remains possible that feral colonies are more able to cope with the observed pathogen load than their managed counterparts.

Given the novel observations that (i) feral colonies contain crippling high levels of DWV; (ii) managed and feral populations appear similar using three different measures of wing morphometry and (iii) feral and pathogen populations share even recently emerged parasites, it seems likely that the invasion of the *Varroa* mite and the increase in prevalence of its concomitant viruses may indeed explain the loss of feral honey bee colonies. Despite showing high levels of DWV in feral colonies, we cannot categorically link this to an increase in feral colony mortality. Future studies could concentrate on understanding whether our observations of high DWV titre result in colony mortality or whether feral populations have behavioural adaptations, such as increased swarming, to tolerate levels of DWV that would be detrimental to a managed colony. Finally, future work could use microsatellite markers to categorically explore the relatedness of feral and managed honey bee populations.

## References

[1] Martin SJ, Highfield AC, Brettell L, Villalobos EM, Budge GE, et al (2012) Global honey bee viral landscape altered by a parasitic mite. Science 336: 1304–1306. 22679096 10.1126/science.1220941

[2] Carreck NL, Ball BV, Wilson JK (2002) Virus succession in honeybee colonies infested with *Varroa destructor* . Apiacta 37: 33–38.

[3] Le Conte Y, Ellis M, Ritter W (2010) *Varroa* mites and honey bee health: can *Varroa* explain part of the colony losses? Apidologie 41: 353–363.

[4] Locke B, Le Conte Y, Crauser D, Fries I (2012) Host adaptations reduce the reproductive success of *Varroa destructor* in two distinct European honey bee populations. Ecol Evol 2: 1144–1150. 22833790 10.1002/ece3.248 PMC3402190

[5] Rosenkranz P, Aumeier P, Ziegelmann B (2010) Biology and control of *Varroa destructor* . J Invertebr Pathol 103 Suppl: S96–119. 19909970 10.1016/j.jip.2009.07.016

[6] Boecking O, Genersch E (2008) Varroosis – the Ongoing Crisis in Bee Keeping. J für Verbraucherschutz und Leb 3: 221–228.

[7] Genersch E, von der Ohe W, Kaatz H, Schroeder A, Otten C, et al (2010) The German bee monitoring project: a long term study to understand periodically high winter losses of honey bee colonies. Apidologie 41: 332–352.

[8] Highfield AC, El Nagar A, Mackinder LCM, Laure MLJN, Hall MJ, et al (2009) Deformed wing virus implicated in overwintering honeybee colony losses. Appl Environ Microbiol 75: 7212–7220. 19783750 10.1128/AEM.02227-09 PMC2786540

[9] Genersch E (2010) Honey bee pathology: current threats to honey bees and beekeeping. Appl Microbiol Biotechnol 87: 87–97. 20401479 10.1007/s00253-010-2573-8

[10] De Miranda JR, Genersch E (2010) Deformed wing virus. J Invertebr Pathol 103 Suppl: S48–61. 19909976 10.1016/j.jip.2009.06.012

[11] Wallner K, Fries I (2003) Control of the mite *Varroa destructor* in honey bee colonies. Pestic Outlook 14: 80–84.

[12] Doebler S (2000) The rise and fall of the honeybee. Bioscience 50: 738–742.

[13] Le Conte Y, Vaublanc G, Crauser D, Jeanne F, Rousselle J, et al (2007) Honey bee colonies that have survived *Varroa destructor* . Apidologie 38: 566–572.

[14] Locke B, Fries I (2011) Characteristics of honey bee colonies (*Apis mellifera*) in Sweden surviving *Varroa destructor* infestation. Apidologie 42: 533–542.

[15] Seeley TD (2007) Honey bees of the Arnot Forest: a population of feral colonies persisting with *Varroa destructor* in the north eastern United States. Apidologie 38: 19–29.

[16] Büchler R, Berg S, Le Conte Y (2010) Breeding for resistance to Varroa destructor in Europe. Apidologie 41: 393–408.

[17] Green D, Kraaijeveld A, Godfray H (2000) Evolutionary interactions between Drosophila melanogaster and its parasitoid Asobara tabida. Heredity (Edinb) 85: 450–458. 11122423 10.1046/j.1365-2540.2000.00788.x

[18] Kerstes NAG, Martin OY (2013) Insect host-parasite coevolution in the light of experimental evolution. Insect Sci 21(4): 401–414. 24130157 10.1111/1744-7917.12064

[19] Villa JD, Rinderer TE (2008) Inheritance of resistance to *Acarapis woodi* (Acari: Tarsonemidae) in crosses between selected resistant Russian and selected susceptible U.S. honey bees (Hymenoptera: Apidae). J Econ Entomol 101: 1756–1759. 19133453 10.1603/0022-0493-101.6.1756

[20] Ratnieks F, Nowakowski J (1989) Honeybee swarms accept hives contaminated with American foulbrood disease. Ecol Entomol 14: 475–478.

[21] Taylor MA, Goodwin RM, McBrydie HM, Cox HM (2007) Destroying managed and feral honey bee (*Apis mellifera*) colonies to eradicate honey bee pests. New Zeal J Crop Hortic Sci 35: 313–323.

[22] Budge G, Adams I, Jones B, Marris G, Laurenson L, et al (2010) Investigating honey bee colony health in England and Wales. FERA 1–2.

[23] Thompson C, Budge G, Biesmeijer J (2010) Feral Bees in the UK: The Real Story. Bee Cr 22–24.

[24] Iqbal J, Mueller U (2007) Virus infection causes specific learning deficits in honeybee foragers. Proc R Soc B 274: 1517–1521. 10.1098/rspb.2007.0022 PMC2176156 17439851

[25] Chang S, Puryear J, Cairney J (1993) A simple and efficient method for isolating RNA from pine trees. Plant Mol Biol Report 11: 113–116.

[26] Ratti C, Budge G, Ward L, Clover G, Rubies-Autonell C, et al (2004) Detection and relative quantitation of soil-borne cereal mosaic virus (SBCMV) and *Polymyxa graminis* in winter wheat using real-time PCR (TaqMan (R)). J Virol Methods 122: 95–103. 15488626 10.1016/j.jviromet.2004.08.013

[27] Schmittgen TD, Livak KJ (2008) Analyzing real-time PCR data by the comparative CT method. Nat Protoc 3: 1101–1108. 18546601 10.1038/nprot.2008.73

[28] Toma DP, Bloch G, Moore D, Robinson GE (2000) Changes in period mRNA levels in the brain and division of labor in honey bee colonies. Proc Natl Acad Sci 97: 6914–6919. 10841583 10.1073/pnas.97.12.6914 PMC18775

[29] Yamazaki Y, Shirai K, Paul RK, Fujiyuki T, Wakamoto A, et al (2006) Differential expression of HR38 in the mushroom bodies of the honeybee brain depends on the caste and division of labour. FEBS Lett 580: 2667–2670. 16647071 10.1016/j.febslet.2006.04.016

[30] Lourenço AP, Mackert A, dos Santos Cristino A, Simões ZLP (2008) Validation of reference genes for gene expression studies in the honey bee, Apis mellifera, by quantitative real-time RT-PCR. Apidologie 39: 372–385.

[31] Livak KJ, Schmittgen T (2001) Analysis of relative gene expression data using real-time quantitative PCR and the 2(-Delta Delta C(T)) Method. Methods 25: 402–408. 11846609 10.1006/meth.2001.1262

[32] Tofilski A (2008) Using geometric morphometrics and standard morphometry to discriminate three honeybee subspecies. Apidologie 39: 558–563.

[33] Tofilski A (2004) DrawWing, a program for numerical description of insect wings. J Insect Sci 4: 4–17. 15861233 10.1093/jis/4.1.17 PMC528877

[34] Edwards P (2007) MorphPlotV2.2. [Computer program] Available: http://www.bibba.com/downloads.php. Accessed 2014 Jul 27.

[35] VSNInternational (2011) GenStat for Windows 14th Edition. Available: https://www.vsni.co.uk/downloads/genstat/14th-edition-upgrade. Accessed 2014 Jul 27.

[36] Hollander M, Wolfe DA (1973) Nonparametric Statistical Methods: John Wiley and Sons.

[37] Bailey L, Ball B V, Perry JN (1981) The prevalence of viruses of honey bees in Britain. Ann Appl Biol 97: 109–118.

[38] Ingemar F, Scott C (2001) Implications of horizontal and vertical pathogen transmission for honey bee epidemiology. Apidologie. 32: 199–214.

[39] Ruttner F (1988) Biogeography and taxonomy of honeybees. : Springer.

[40] Moritz RFA (1991) The limitations of biometric control on pure race breeding in *Apis mellifera* . J Apic Res 30: 54–59.

[41] Bouga M, Alaux C, Bienkowska M, Büchler R, Carreck NL, et al (2011) A review of methods for discrimination of honey bee populations as applied to European beekeeping. J Apic Res 50: 51–84.

[42] DEFRA (2013) Bees and other pollinators: their value and health in England. Available: http://www.step-project.net/files/DOWNLOAD2/pb13981-bees-pollinators-review.pdf. Accessed 2014 Jul 27.

[43] Fries I, Bommarco R (2007) Original article possible host-parasite adaptations in honey bees infested by *Varroa destructor* mites. Apidologie 38: 525–533.

[44] Dietemann V, Neumann P, Ellis J (2013) Standard methods for varroa research. COLOSS BEEBOOK, Vol II Stand methods *Apis mellifera* pest. Pathog Res 52 (1): 1–54.

[45] Paxton RJ, Klee J, Korpela S, Fries I (2007) *Nosema ceranae* has infected *Apis mellifera* in Europe since at least 1998 and may be more virulent than *Nosema apis* . Apidologie 38: 558–565.

[46] Evison SEF, Roberts KE, Laurenson L, Pietravalle S, Hui J, et al (2012) Pervasiveness of parasites in pollinators. PLoS One 7: 1–7. 10.1371/journal.pone.0030641 PMC3273957 22347356

[47] Bailey L (1961) European foulbrood. Am bee J 101: 89–92.

[48] Budge GE, Barrett B, Jones B, Pietravalle S, Marris G, et al (2010) The occurrence of *Melissococcus plutonius* in healthy colonies of *Apis mellifera* and the efficacy of European foulbrood control measures. J Invertebr Pathol 105: 164–170. 20600088 10.1016/j.jip.2010.06.004

[49] Chantawannakul P, Ward L, Boonham N, Brown MA (2006) A scientific note on the detection of honeybee viruses using real-time PCR (TaqMan) in *Varroa* mites collected from a Tai honeybee (*Apis mellifera*) apiary. J Invertebr Pathol 91: 69–73. 16376930 10.1016/j.jip.2005.11.001

[50] de Miranda JR (2010) Dainat B, Locke B, Cordoni G, Berthoud H, et al (2010) Genetic characterisation of slow paralysis virus of the honeybee (*Apis mellifera*). J Genet Virology 91: 2524–2530. 10.1099/vir.0.022434-0 20519455

[51] Budge GE, Pietravalle S, Brown M, Laurenson L, Jones B, et al (2014) Pathogens as predictors of colony strength in England and Wales. . PLoS One In press 10.1371/journal.pone.0133228 PMC4506140 26186735

